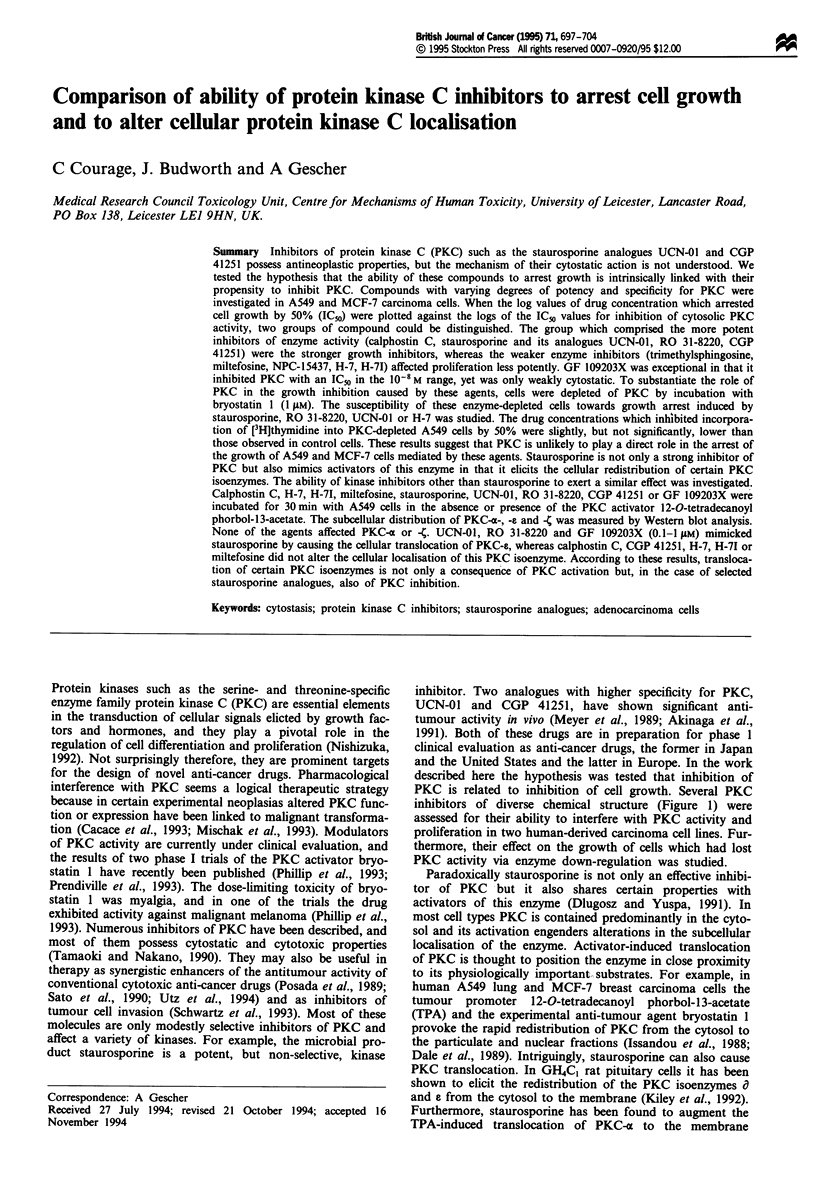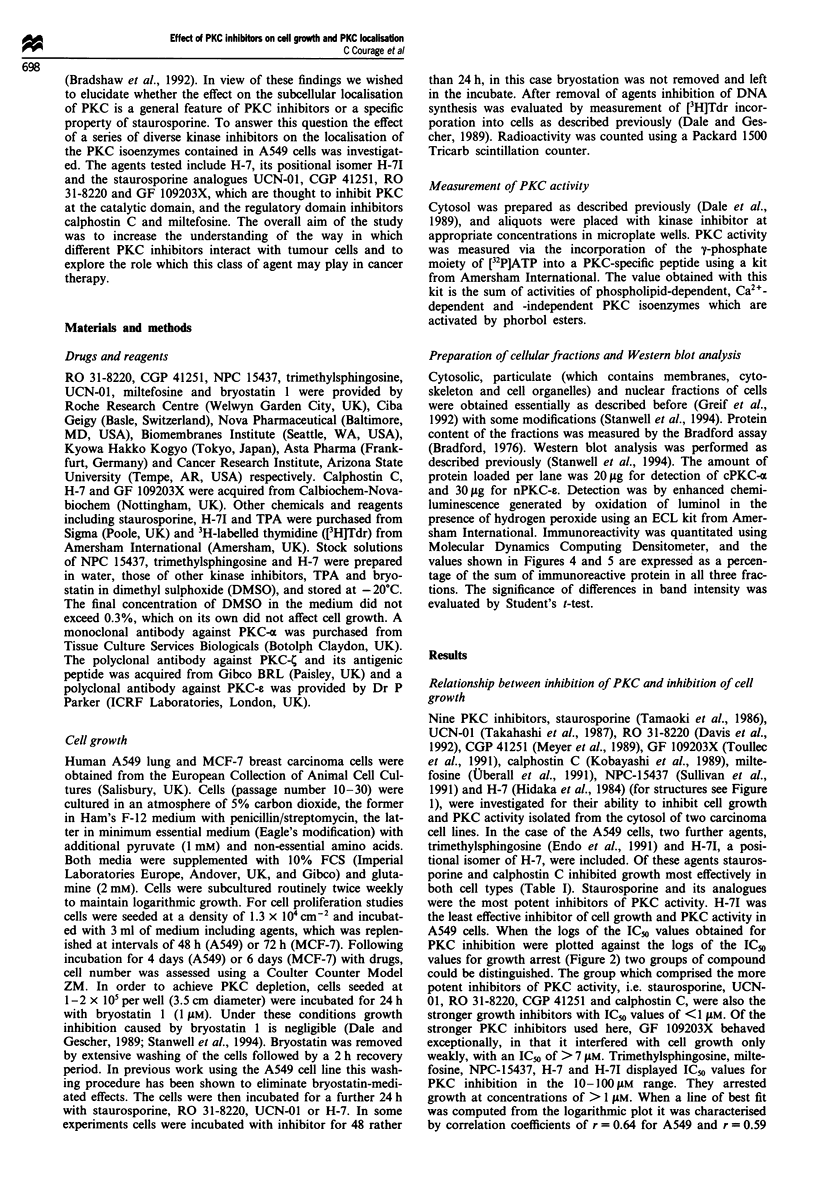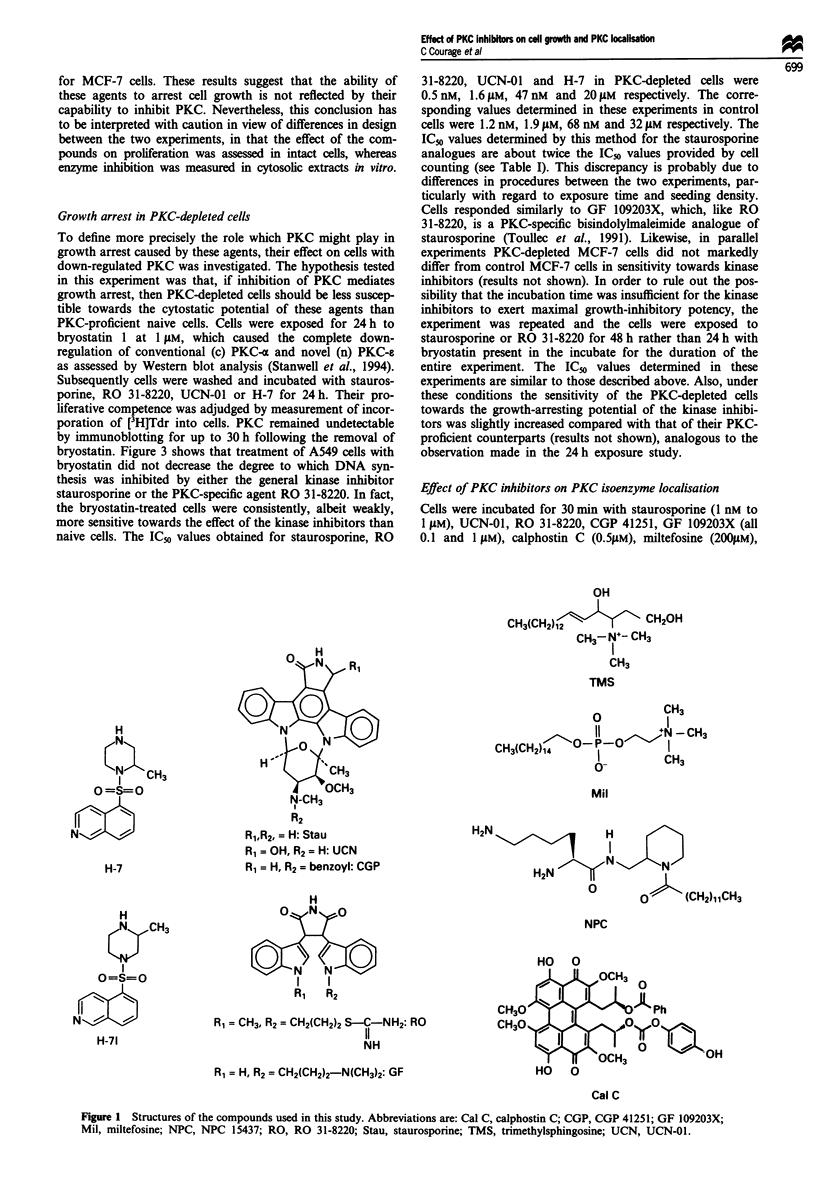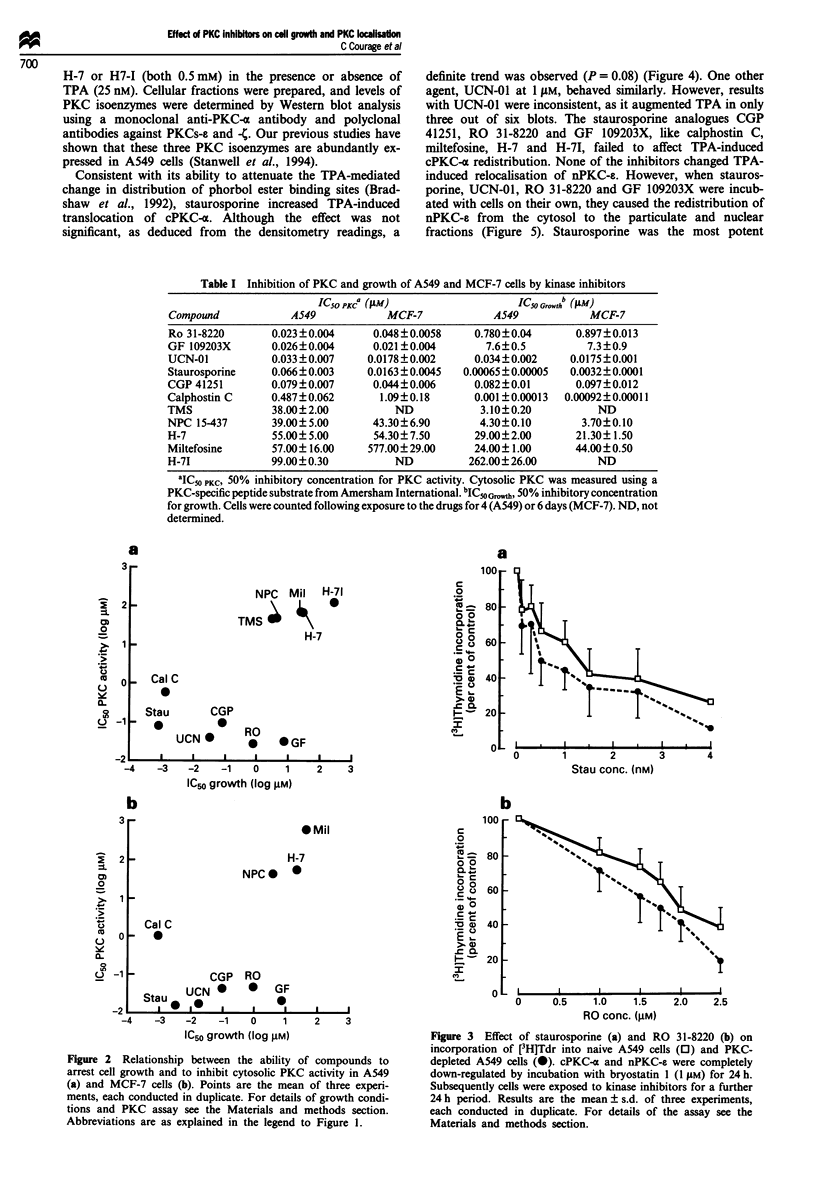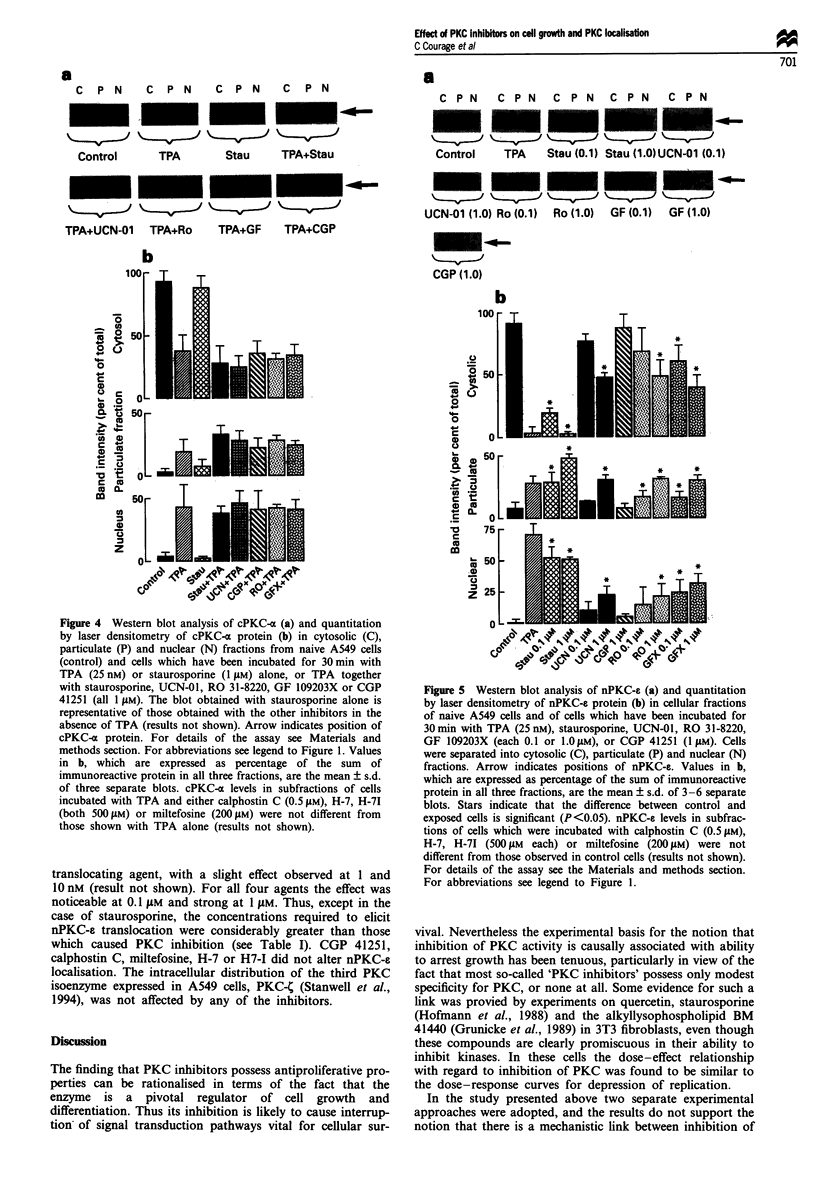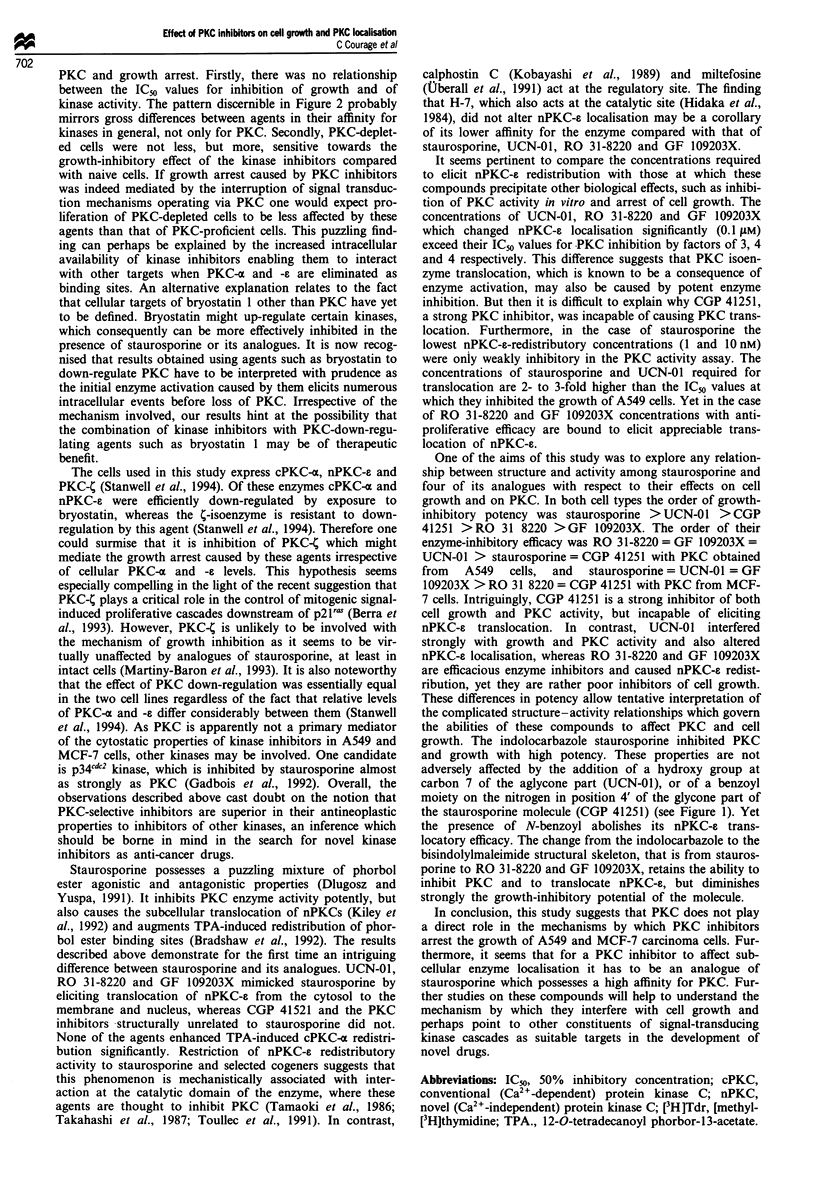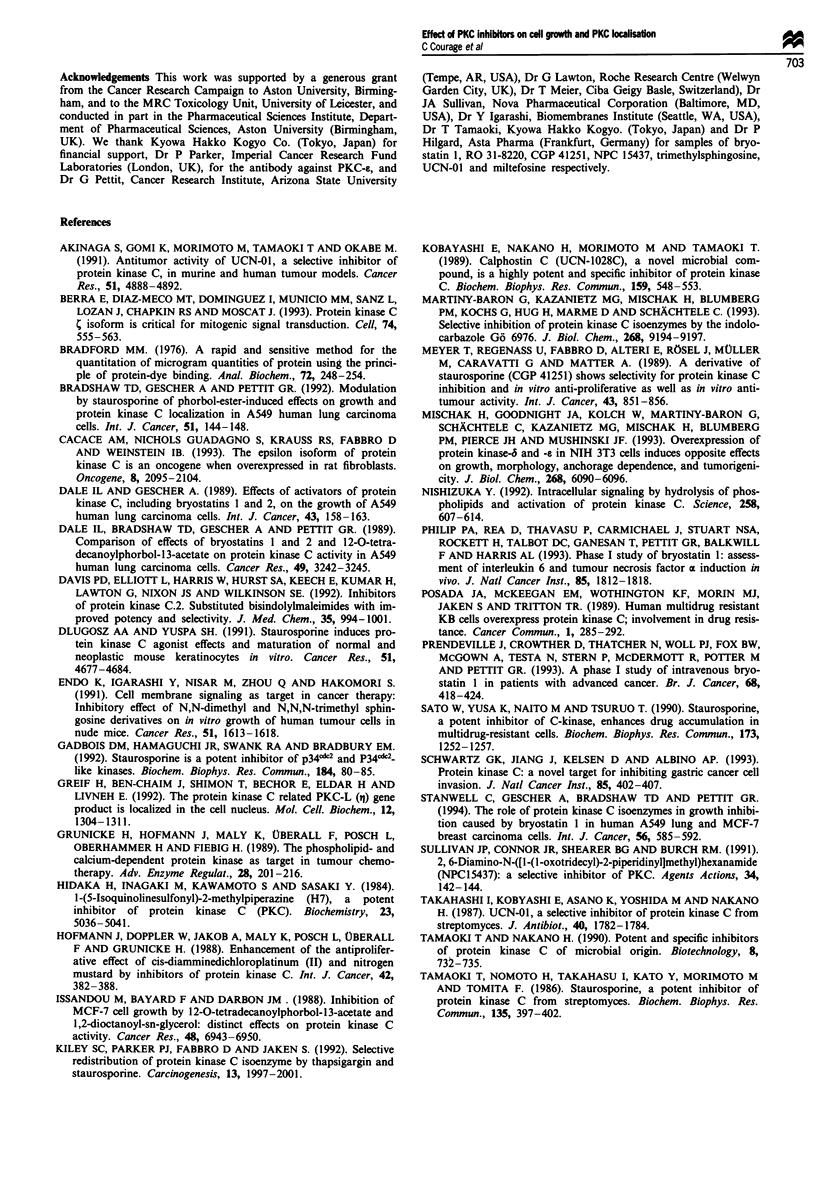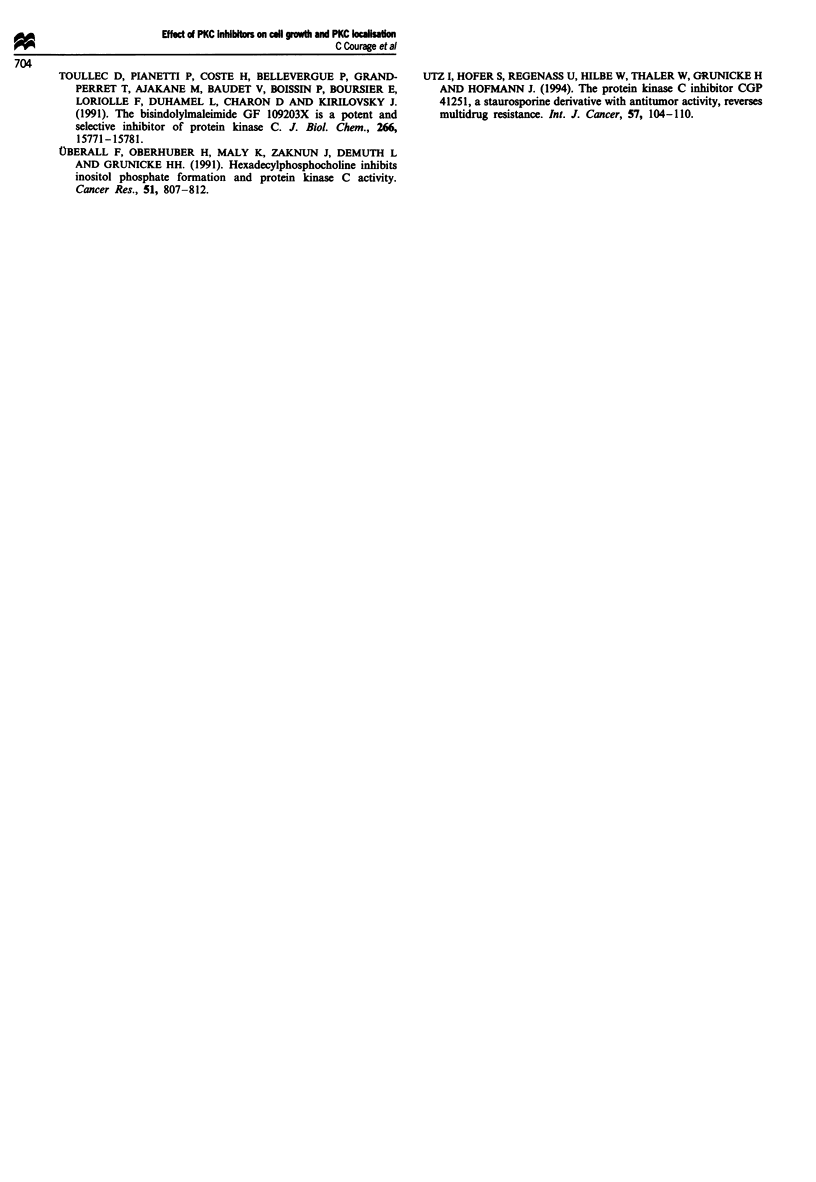# Comparison of ability of protein kinase C inhibitors to arrest cell growth and to alter cellular protein kinase C localisation.

**DOI:** 10.1038/bjc.1995.137

**Published:** 1995-04

**Authors:** C. Courage, J. Budworth, A. Gescher

**Affiliations:** Medical Research Council Toxicology Unit, University of Leicester, UK.

## Abstract

**Images:**


British Journal of Cancer (1995) 71, 697–704
© 1995 Stockton Press All rights reserved 0007–0920/95 $12.00

### Comparison of ability of protein kinase C inhibitors to arrest cell growth and to alter cellular protein kinase C localisation

C Courage, J. Budworth and A Gescher

*Medical Research Council Toxicology Unit, Centre for Mechanisms of Human Toxicity, University of Leicester, Lancaster Road, PO Box 138, Leicester LE1 9HN, UK.*

**Summary** Inhibitors of protein kinase C (PKC) such as the staurosporine analogues UCN-01 and CGP 41251 possess antineoplastic properties, but the mechanism of their cytostatic action is not understood. We tested the hypothesis that the ability of these compounds to arrest growth is intrinsically linked with their propensity to inhibit PKC. Compounds with varying degrees of potency and specificity for PKC were investigated in A549 and MCF-7 carcinoma cells. When the log values of drug concentration which arrested cell growth by 50% ($IC_{50}$) were plotted against the logs of the $IC_{50}$ values for inhibition of cytosolic PKC activity, two groups of compound could be distinguished. The group which comprised the more potent inhibitors of enzyme activity (calphostin C, staurosporine and its analogues UCN-01, RO 31-8220, CGP 41251) were the stronger growth inhibitors, whereas the weaker enzyme inhibitors (trimethylsphingosine, miltefosine, NPC-15437, H-7, H-7I) affected proliferation less potently. GF 109203X was exceptional in that it inhibited PKC with an $IC_{50}$ in the $10^{-8}$ M range, yet was only weakly cytostatic. To substantiate the role of PKC in the growth inhibition caused by these agents, cells were depleted of PKC by incubation with bryostatin 1 (1 μM). The susceptibility of these enzyme-depleted cells towards growth arrest induced by staurosporine, RO 31-8220, UCN-01 or H-7 was studied. The drug concentrations which inhibited incorporation of [$^3$H]thymidine into PKC-depleted A549 cells by 50% were slightly, but not significantly, lower than those observed in control cells. These results suggest that PKC is unlikely to play a direct role in the arrest of the growth of A549 and MCF-7 cells mediated by these agents. Staurosporine is not only a strong inhibitor of PKC but also mimics activators of this enzyme in that it elicits the cellular redistribution of certain PKC isoenzymes. The ability of kinase inhibitors other than staurosporine to exert a similar effect was investigated. Calphostin C, H-7, H-7I, miltefosine, staurosporine, UCN-01, RO 31-8220, CGP 41251 or GF 109203X were incubated for 30 min with A549 cells in the absence or presence of the PKC activator 12-*O*-tetradecanoyl phorbol-13-acetate. The subcellular distribution of PKC-α-, -ε and -ζ was measured by Western blot analysis. None of the agents affected PKC-α or -ζ. UCN-01, RO 31-8220 and GF 109203X (0.1–1 μM) mimicked staurosporine by causing the cellular translocation of PKC-ε, whereas calphostin C, CGP 41251, H-7, H-7I or miltefosine did not alter the cellular localisation of this PKC isoenzyme. According to these results, translocation of certain PKC isoenzymes is not only a consequence of PKC activation but, in the case of selected staurosporine analogues, also of PKC inhibition.

**Keywords:** cytostasis; protein kinase C inhibitors; staurosporine analogues; adenocarcinoma cells

Protein kinases such as the serine- and threonine-specific enzyme family protein kinase C (PKC) are essential elements in the transduction of cellular signals elicted by growth factors and hormones, and they play a pivotal role in the regulation of cell differentiation and proliferation (Nishizuka, 1992). Not surprisingly therefore, they are prominent targets for the design of novel anti-cancer drugs. Pharmacological interference with PKC seems a logical therapeutic strategy because in certain experimental neoplasias altered PKC function or expression have been linked to malignant transformation (Cacace *et al.*, 1993; Mischak *et al.*, 1993). Modulators of PKC activity are currently under clinical evaluation, and the results of two phase I trials of the PKC activator bryostatin 1 have recently been published (Phillip *et al.*, 1993; Prendiville *et al.*, 1993). The dose-limiting toxicity of bryostatin 1 was myalgia, and in one of the trials the drug exhibited activity against malignant melanoma (Phillip *et al.*, 1993). Numerous inhibitors of PKC have been described, and most of them possess cytostatic and cytotoxic properties (Tamaoki and Nakano, 1990). They may also be useful in therapy as synergistic enhancers of the antitumour activity of conventional cytotoxic anti-cancer drugs (Posada *et al.*, 1989; Sato *et al.*, 1990; Utz *et al.*, 1994) and as inhibitors of tumour cell invasion (Schwartz *et al.*, 1993). Most of these molecules are only modestly selective inhibitors of PKC and affect a variety of kinases. For example, the microbial product staurosporine is a potent, but non-selective, kinase inhibitor. Two analogues with higher specificity for PKC, UCN-01 and CGP 41251, have shown significant antitumour activity *in vivo* (Meyer *et al.*, 1989; Akinaga *et al.*, 1991). Both of these drugs are in preparation for phase 1 clinical evaluation as anti-cancer drugs, the former in Japan and the United States and the latter in Europe. In the work described here the hypothesis was tested that inhibition of PKC is related to inhibition of cell growth. Several PKC inhibitors of diverse chemical structure (Figure 1) were assessed for their ability to interfere with PKC activity and proliferation in two human-derived carcinoma cell lines. Furthermore, their effect on the growth of cells which had lost PKC activity via enzyme down-regulation was studied.

Paradoxically staurosporine is not only an effective inhibitor of PKC but it also shares certain properties with activators of this enzyme (Dlugosz and Yuspa, 1991). In most cell types PKC is contained predominantly in the cytosol and its activation engenders alterations in the subcellular localisation of the enzyme. Activator-induced translocation of PKC is thought to position the enzyme in close proximity to its physiologically important substrates. For example, in human A549 lung and MCF-7 breast carcinoma cells the tumour promoter 12-*O*-tetradecanoyl phorbol-13-acetate (TPA) and the experimental anti-tumour agent bryostatin 1 provoke the rapid redistribution of PKC from the cytosol to the particulate and nuclear fractions (Issandou *et al.*, 1988; Dale *et al.*, 1989). Intriguingly, staurosporine can also cause PKC translocation. In $GH_4C_1$ rat pituitary cells it has been shown to elicit the redistribution of the PKC isoenzymes ∂ and ε from the cytosol to the membrane (Kiley *et al.*, 1992). Furthermore, staurosporine has been found to augment the TPA-induced translocation of PKC-α to the membrane

Correspondence: A Gescher
Received 27 July 1994; revised 21 October 1994; accepted 16 November 1994

(Bradshaw et al., 1992). In view of these findings we wished to elucidate whether the effect on the subcellular localisation of PKC is a general feature of PKC inhibitors or a specific property of staurosporine. To answer this question the effect of a series of diverse kinase inhibitors on the localisation of the PKC isoenzymes contained in A549 cells was investigated. The agents tested include H-7, its positional isomer H-7I and the staurosporine analogues UCN-01, CGP 41251, RO 31-8220 and GF 109203X, which are thought to inhibit PKC at the catalytic domain, and the regulatory domain inhibitors calphostin C and miltefosine. The overall aim of the study was to increase the understanding of the way in which different PKC inhibitors interact with tumour cells and to explore the role which this class of agent may play in cancer therapy.

### Materials and methods

### *Drugs and reagents*

RO 31-8220, CGP 41251, NPC 15437, trimethylsphingosine, UCN-01, miltefosine and bryostatin 1 were provided by Roche Research Centre (Welwyn Garden City, UK), Ciba Geigy (Basle, Switzerland), Nova Pharmaceutical (Baltimore, MD, USA), Biomembranes Institute (Seattle, WA, USA), Kyowa Hakko Kogyo (Tokyo, Japan), Asta Pharma (Frankfurt, Germany) and Cancer Research Institute, Arizona State University (Tempe, AR, USA) respectively. Calphostin C, H-7 and GF 109203X were acquired from Calbiochem-Novabiochem (Nottingham, UK). Other chemicals and reagents including staurosporine, H-7I and TPA were purchased from Sigma (Poole, UK) and $^3$H-labelled thymidine ([$^3$H]Tdr) from Amersham International (Amersham, UK). Stock solutions of NPC 15437, trimethylsphingosine and H-7 were prepared in water, those of other kinase inhibitors, TPA and bryostatin in dimethyl sulphoxide (DMSO), and stored at $-20$°C. The final concentration of DMSO in the medium did not exceed 0.3%, which on its own did not affect cell growth. A monoclonal antibody against PKC-α was purchased from Tissue Culture Services Biologicals (Botolph Claydon, UK). The polyclonal antibody against PKC-ζ and its antigenic peptide was acquired from Gibco BRL (Paisley, UK) and a polyclonal antibody against PKC-ε was provided by Dr P Parker (ICRF Laboratories, London, UK).

### *Cell growth*

Human A549 lung and MCF-7 breast carcinoma cells were obtained from the European Collection of Animal Cell Cultures (Salisbury, UK). Cells (passage number 10–30) were cultured in an atmosphere of 5% carbon dioxide, the former in Ham's F-12 medium with penicillin/streptomycin, the latter in minimum essential medium (Eagle's modification) with additional pyruvate (1 mM) and non-essential amino acids. Both media were supplemented with 10% FCS (Imperial Laboratories Europe, Andover, UK, and Gibco) and glutamine (2 mM). Cells were subcultured routinely twice weekly to maintain logarithmic growth. For cell proliferation studies cells were seeded at a density of $1.3 \times 10^4$ cm$^{-2}$ and incubated with 3 ml of medium including agents, which was replenished at intervals of 48 h (A549) or 72 h (MCF-7). Following incubation for 4 days (A549) or 6 days (MCF-7) with drugs, cell number was assessed using a Coulter Counter Model ZM. In order to achieve PKC depletion, cells seeded at $1-2 \times 10^5$ per well (3.5 cm diameter) were incubated for 24 h with bryostatin 1 (1 μM). Under these conditions growth inhibition caused by bryostatin 1 is negligible (Dale and Gescher, 1989; Stanwell et al., 1994). Bryostatin was removed by extensive washing of the cells followed by a 2 h recovery period. In previous work using the A549 cell line this washing procedure has been shown to eliminate bryostatin-mediated effects. The cells were then incubated for a further 24 h with staurosporine, RO 31-8220, UCN-01 or H-7. In some experiments cells were incubated with inhibitor for 48 rather than 24 h, in this case bryostation was not removed and left in the incubate. After removal of agents inhibition of DNA synthesis was evaluated by measurement of [$^3$H]Tdr incorporation into cells as described previously (Dale and Gescher, 1989). Radioactivity was counted using a Packard 1500 Tricarb scintillation counter.

### *Measurement of PKC activity*

Cytosol was prepared as described previously (Dale et al., 1989), and aliquots were placed with kinase inhibitor at appropriate concentrations in microplate wells. PKC activity was measured via the incorporation of the γ-phosphate moiety of [$^{32}$P]ATP into a PKC-specific peptide using a kit from Amersham International. The value obtained with this kit is the sum of activities of phospholipid-dependent, $Ca^{2+}$-dependent and -independent PKC isoenzymes which are activated by phorbol esters.

### *Preparation of cellular fractions and Western blot analysis*

Cytosolic, particulate (which contains membranes, cytoskeleton and cell organelles) and nuclear fractions of cells were obtained essentially as described before (Greif et al., 1992) with some modifications (Stanwell et al., 1994). Protein content of the fractions was measured by the Bradford assay (Bradford, 1976). Western blot analysis was performed as described previously (Stanwell et al., 1994). The amount of protein loaded per lane was 20 μg for detection of cPKC-α and 30 μg for nPKC-ε. Detection was by enhanced chemiluminescence generated by oxidation of luminol in the presence of hydrogen peroxide using an ECL kit from Amersham International. Immunoreactivity was quantitated using Molecular Dynamics Computing Densitometer, and the values shown in Figures 4 and 5 are expressed as a percentage of the sum of immunoreactive protein in all three fractions. The significance of differences in band intensity was evaluated by Student's t-test.

### Results

### *Relationship between inhibition of PKC and inhibition of cell growth*

Nine PKC inhibitors, staurosporine (Tamaoki et al., 1986), UCN-01 (Takahashi et al., 1987), RO 31-8220 (Davis et al., 1992), CGP 41251 (Meyer et al., 1989), GF 109203X (Toullec et al., 1991), calphostin C (Kobayashi et al., 1989), miltefosine (Überall et al., 1991), NPC-15437 (Sullivan et al., 1991) and H-7 (Hidaka et al., 1984) (for structures see Figure 1), were investigated for their ability to inhibit cell growth and PKC activity isolated from the cytosol of two carcinoma cell lines. In the case of the A549 cells, two further agents, trimethylsphingosine (Endo et al., 1991) and H-7I, a positional isomer of H-7, were included. Of these agents staurosporine and calphostin C inhibited growth most effectively in both cell types (Table I). Staurosporine and its analogues were the most potent inhibitors of PKC activity. H-7I was the least effective inhibitor of cell growth and PKC activity in A549 cells. When the logs of the $IC_{50}$ values obtained for PKC inhibition were plotted against the logs of the $IC_{50}$ values for growth arrest (Figure 2) two groups of compound could be distinguished. The group which comprised the more potent inhibitors of PKC activity, i.e. staurosporine, UCN-01, RO 31-8220, CGP 41251 and calphostin C, were also the stronger growth inhibitors with $IC_{50}$ values of $<1$ μM. Of the stronger PKC inhibitors used here, GF 109203X behaved exceptionally, in that it interfered with cell growth only weakly, with an $IC_{50}$ of $>7$ μM. Trimethylsphingosine, miltefosine, NPC-15437, H-7 and H-7I displayed $IC_{50}$ values for PKC inhibition in the 10–100 μM range. They arrested growth at concentrations of $>1$ μM. When a line of best fit was computed from the logarithmic plot it was characterised by correlation coefficients of $r = 0.64$ for A549 and $r = 0.59$

for MCF-7 cells. These results suggest that the ability of these agents to arrest cell growth is not reflected by their capability to inhibit PKC. Nevertheless, this conclusion has to be interpreted with caution in view of differences in design between the two experiments, in that the effect of the compounds on proliferation was assessed in intact cells, whereas enzyme inhibition was measured in cytosolic extracts *in vitro*.

### *Growth arrest in PKC-depleted cells*

To define more precisely the role which PKC might play in growth arrest caused by these agents, their effect on cells with down-regulated PKC was investigated. The hypothesis tested in this experiment was that, if inhibition of PKC mediates growth arrest, then PKC-depleted cells should be less susceptible towards the cytostatic potential of these agents than PKC-proficient naive cells. Cells were exposed for 24 h to bryostatin 1 at 1 μM, which caused the complete down-regulation of conventional (c) PKC-α and novel (n) PKC-ε as assessed by Western blot analysis (Stanwell *et al.*, 1994). Subsequently cells were washed and incubated with staurosporine, RO 31-8220, UCN-01 or H-7 for 24 h. Their proliferative competence was adjudged by measurement of incorporation of [$^3$H]Tdr into cells. PKC remained undetectable by immunoblotting for up to 30 h following the removal of bryostatin. Figure 3 shows that treatment of A549 cells with bryostatin did not decrease the degree to which DNA synthesis was inhibited by either the general kinase inhibitor staurosporine or the PKC-specific agent RO 31-8220. In fact, the bryostatin-treated cells were consistently, albeit weakly, more sensitive towards the effect of the kinase inhibitors than naive cells. The $IC_{50}$ values obtained for staurosporine, RO 31-8220, UCN-01 and H-7 in PKC-depleted cells were 0.5 nM, 1.6 μM, 47 nM and 20 μM respectively. The corresponding values determined in these experiments in control cells were 1.2 nM, 1.9 μM, 68 nM and 32 μM respectively. The $IC_{50}$ values determined by this method for the staurosporine analogues are about twice the $IC_{50}$ values provided by cell counting (see Table I). This discrepancy is probably due to differences in procedures between the two experiments, particularly with regard to exposure time and seeding density. Cells responded similarly to GF 109203X, which, like RO 31-8220, is a PKC-specific bisindolylmaleimide analogue of staurosporine (Toullec *et al.*, 1991). Likewise, in parallel experiments PKC-depleted MCF-7 cells did not markedly differ from control MCF-7 cells in sensitivity towards kinase inhibitors (results not shown). In order to rule out the possibility that the incubation time was insufficient for the kinase inhibitors to exert maximal growth-inhibitory potency, the experiment was repeated and the cells were exposed to staurosporine or RO 31-8220 for 48 h rather than 24 h with bryostatin present in the incubate for the duration of the entire experiment. The $IC_{50}$ values determined in these experiments are similar to those described above. Also, under these conditions the sensitivity of the PKC-depleted cells towards the growth-arresting potential of the kinase inhibitors was slightly increased compared with that of their PKC-proficient counterparts (results not shown), analogous to the observation made in the 24 h exposure study.

### *Effect of PKC inhibitors on PKC isoenzyme localisation*

Cells were incubated for 30 min with staurosporine (1 nM to 1 μM), UCN-01, RO 31-8220, CGP 41251, GF 109203X (all 0.1 and 1 μM), calphostin C (0.5μM), miltefosine (200μM),

**Figure 1** Structures of the compounds used in this study. Abbreviations are: Cal C, calphostin C; CGP, CGP 41251; GF 109203X; Mil, miltefosine; NPC, NPC 15437; RO, RO 31-8220; Stau, staurosporine; TMS, trimethylsphingosine; UCN, UCN-01.

H-7 or H7-I (both 0.5 mM) in the presence or absence of TPA (25 nM). Cellular fractions were prepared, and levels of PKC isoenzymes were determined by Western blot analysis using a monoclonal anti-PKC-α antibody and polyclonal antibodies against PKCs-ε and -ζ. Our previous studies have shown that these three PKC isoenzymes are abundantly expressed in A549 cells (Stanwell *et al.*, 1994).

Consistent with its ability to attenuate the TPA-mediated change in distribution of phorbol ester binding sites (Bradshaw *et al.*, 1992), staurosporine increased TPA-induced translocation of cPKC-α. Although the effect was not significant, as deduced from the densitometry readings, a definite trend was observed ($P = 0.08$) (Figure 4). One other agent, UCN-01 at 1 μM, behaved similarly. However, results with UCN-01 were inconsistent, as it augmented TPA in only three out of six blots. The staurosporine analogues CGP 41251, RO 31-8220 and GF 109203X, like calphostin C, miltefosine, H-7 and H-7I, failed to affect TPA-induced cPKC-α redistribution. None of the inhibitors changed TPA-induced relocalisation of nPKC-ε. However, when staurosporine, UCN-01, RO 31-8220 and GF 109203X were incubated with cells on their own, they caused the redistribution of nPKC-ε from the cytosol to the particulate and nuclear fractions (Figure 5). Staurosporine was the most potent

**Table I** Inhibition of PKC and growth of A549 and MCF-7 cells by kinase inhibitors

| | $IC_{50\ PKC}$[a] (μM) | | $IC_{50\ Growth}$[b] (μM) | |
|---|---|---|---|---|
| *Compound* | *A549* | *MCF-7* | *A549* | *MCF-7* |
| Ro 31-8220 | 0.023±0.004 | 0.048±0.0058 | 0.780±0.04 | 0.897±0.013 |
| GF 109203X | 0.026±0.004 | 0.021±0.004 | 7.6±0.5 | 7.3±0.9 |
| UCN-01 | 0.033±0.007 | 0.0178±0.002 | 0.034±0.002 | 0.0175±0.001 |
| Staurosporine | 0.066±0.003 | 0.0163±0.0045 | 0.00065±0.00005 | 0.0032±0.0001 |
| CGP 41251 | 0.079±0.007 | 0.044±0.006 | 0.082±0.01 | 0.097±0.012 |
| Calphostin C | 0.487±0.062 | 1.09±0.18 | 0.001±0.00013 | 0.00092±0.00011 |
| TMS | 38.00±2.00 | ND | 3.10±0.20 | ND |
| NPC 15-437 | 39.00±5.00 | 43.30±6.90 | 4.30±0.10 | 3.70±0.10 |
| H-7 | 55.00±5.00 | 54.30±7.50 | 29.00±2.00 | 21.30±1.50 |
| Miltefosine | 57.00±16.00 | 577.00±29.00 | 24.00±1.00 | 44.00±0.50 |
| H-7I | 99.00±0.30 | ND | 262.00±26.00 | ND |

[a]$IC_{50\ PKC}$, 50% inhibitory concentration for PKC activity. Cytosolic PKC was measured using a PKC-specific peptide substrate from Amersham International. [b]$IC_{50\ Growth}$, 50% inhibitory concentration for growth. Cells were counted following exposure to the drugs for 4 (A549) or 6 days (MCF-7). ND, not determined.

**Figure 2** Relationship between the ability of compounds to arrest cell growth and to inhibit cytosolic PKC activity in A549 (**a**) and MCF-7 cells (**b**). Points are the mean of three experiments, each conducted in duplicate. For details of growth conditions and PKC assay see the Materials and methods section. Abbreviations are as explained in the legend to Figure 1.

**Figure 3** Effect of staurosporine (**a**) and RO 31-8220 (**b**) on incorporation of [$^3$H]Tdr into naive A549 cells (□) and PKC-depleted A549 cells (●). cPKC-α and nPKC-ε were completely down-regulated by incubation with bryostatin 1 (1 μM) for 24 h. Subsequently cells were exposed to kinase inhibitors for a further 24 h period. Results are the mean ± s.d. of three experiments, each conducted in duplicate. For details of the assay see the Materials and methods section.

**Figure 4** Western blot analysis of cPKC-α (**a**) and quantitation by laser densitometry of cPKC-α protein (**b**) in cytosolic (C), particulate (P) and nuclear (N) fractions from naive A549 cells (control) and cells which have been incubated for 30 min with TPA (25 nM) or staurosporine (1 μM) alone, or TPA together with staurosporine, UCN-01, RO 31-8220, GF 109203X or CGP 41251 (all 1 μM). The blot obtained with staurosporine alone is representative of those obtained with the other inhibitors in the absence of TPA (results not shown). Arrow indicates position of cPKC-α protein. For details of the assay see Materials and methods section. For abbreviations see legend to Figure 1. Values in **b**, which are expressed as percentage of the sum of immunoreactive protein in all three fractions, are the mean ± s.d. of three separate blots. cPKC-α levels in subfractions of cells incubated with TPA and either calphostin C (0.5 μM), H-7, H-7I (both 500 μM) or miltefosine (200 μM) were not different from those shown with TPA alone (results not shown).

**Figure 5** Western blot analysis of nPKC-ε (**a**) and quantitation by laser densitometry of nPKC-ε protein (**b**) in cellular fractions of naive A549 cells and of cells which have been incubated for 30 min with TPA (25 nM), staurosporine, UCN-01, RO 31-8220, GF 109203X (each 0.1 or 1.0 μM), or CGP 41251 (1 μM). Cells were separated into cytosolic (C), particulate (P) and nuclear (N) fractions. Arrow indicates positions of nPKC-ε. Values in **b**, which are expressed as percentage of the sum of immunoreactive protein in all three fractions, are the mean ± s.d. of 3–6 separate blots. Stars indicate that the difference between control and exposed cells is significant ($P<0.05$). nPKC-ε levels in subfractions of cells which were incubated with calphostin C (0.5 μM), H-7, H-7I (500 μM each) or miltefosine (200 μM) were not different from those observed in control cells (results not shown). For details of the assay see the Materials and methods section. For abbreviations see legend to Figure 1.

translocating agent, with a slight effect observed at 1 and 10 nM (result not shown). For all four agents the effect was noticeable at 0.1 μM and strong at 1 μM. Thus, except in the case of staurosporine, the concentrations required to elicit nPKC-ε translocation were considerably greater than those which caused PKC inhibition (see Table I). CGP 41251, calphostin C, miltefosine, H-7 or H7-I did not alter nPKC-ε localisation. The intracellular distribution of the third PKC isoenzyme expressed in A549 cells, PKC-ζ (Stanwell *et al.*, 1994), was not affected by any of the inhibitors.

### Discussion

The finding that PKC inhibitors possess antiproliferative properties can be rationalised in terms of the fact that the enzyme is a pivotal regulator of cell growth and differentiation. Thus its inhibition is likely to cause interruption of signal transduction pathways vital for cellular survival. Nevertheless the experimental basis for the notion that inhibition of PKC activity is causally associated with ability to arrest growth has been tenuous, particularly in view of the fact that most so-called 'PKC inhibitors' possess only modest specificity for PKC, or none at all. Some evidence for such a link was provied by experiments on quercetin, staurosporine (Hofmann *et al.*, 1988) and the alkyllysophospholipid BM 41440 (Grunicke *et al.*, 1989) in 3T3 fibroblasts, even though these compounds are clearly promiscuous in their ability to inhibit kinases. In these cells the dose–effect relationship with regard to inhibition of PKC was found to be similar to the dose–response curves for depression of replication.

In the study presented above two separate experimental approaches were adopted, and the results do not support the notion that there is a mechanistic link between inhibition of

PKC and growth arrest. Firstly, there was no relationship between the $IC_{50}$ values for inhibition of growth and of kinase activity. The pattern discernible in Figure 2 probably mirrors gross differences between agents in their affinity for kinases in general, not only for PKC. Secondly, PKC-depleted cells were not less, but more, sensitive towards the growth-inhibitory effect of the kinase inhibitors compared with naive cells. If growth arrest caused by PKC inhibitors was indeed mediated by the interruption of signal transduction mechanisms operating via PKC one would expect proliferation of PKC-depleted cells to be less affected by these agents than that of PKC-proficient cells. This puzzling finding can perhaps be explained by the increased intracellular availability of kinase inhibitors enabling them to interact with other targets when PKC-α and -ε are eliminated as binding sites. An alternative explanation relates to the fact that cellular targets of bryostatin 1 other than PKC have yet to be defined. Bryostatin might up-regulate certain kinases, which consequently can be more effectively inhibited in the presence of staurosporine or its analogues. It is now recognised that results obtained using agents such as bryostatin to down-regulate PKC have to be interpreted with prudence as the initial enzyme activation caused by them elicits numerous intracellular events before loss of PKC. Irrespective of the mechanism involved, our results hint at the possibility that the combination of kinase inhibitors with PKC-down-regulating agents such as bryostatin 1 may be of therapeutic benefit.

The cells used in this study express cPKC-α, nPKC-ε and PKC-ζ (Stanwell *et al.*, 1994). Of these enzymes cPKC-α and nPKC-ε were efficiently down-regulated by exposure to bryostatin, whereas the ζ-isoenzyme is resistant to down-regulation by this agent (Stanwell *et al.*, 1994). Therefore one could surmise that it is inhibition of PKC-ζ which might mediate the growth arrest caused by these agents irrespective of cellular PKC-α and -ε levels. This hypothesis seems especially compelling in the light of the recent suggestion that PKC-ζ plays a critical role in the control of mitogenic signal-induced proliferative cascades downstream of $p21^{ras}$ (Berra *et al.*, 1993). However, PKC-ζ is unlikely to be involved with the mechanism of growth inhibition as it seems to be virtually unaffected by analogues of staurosporine, at least in intact cells (Martiny-Baron *et al.*, 1993). It is also noteworthy that the effect of PKC down-regulation was essentially equal in the two cell lines regardless of the fact that relative levels of PKC-α and -ε differ considerably between them (Stanwell *et al.*, 1994). As PKC is apparently not a primary mediator of the cytostatic properties of kinase inhibitors in A549 and MCF-7 cells, other kinases may be involved. One candidate is $p34^{cdc2}$ kinase, which is inhibited by staurosporine almost as strongly as PKC (Gadbois *et al.*, 1992). Overall, the observations described above cast doubt on the notion that PKC-selective inhibitors are superior in their antineoplastic properties to inhibitors of other kinases, an inference which should be borne in mind in the search for novel kinase inhibitors as anti-cancer drugs.

Staurosporine possesses a puzzling mixture of phorbol ester agonistic and antagonistic properties (Dlugosz and Yuspa, 1991). It inhibits PKC enzyme activity potently, but also causes the subcellular translocation of nPKCs (Kiley *et al.*, 1992) and augments TPA-induced redistribution of phorbol ester binding sites (Bradshaw *et al.*, 1992). The results described above demonstrate for the first time an intriguing difference between staurosporine and its analogues. UCN-01, RO 31-8220 and GF 109203X mimicked staurosporine by eliciting translocation of nPKC-ε from the cytosol to the membrane and nucleus, whereas CGP 41521 and the PKC inhibitors structurally unrelated to staurosporine did not. None of the agents enhanced TPA-induced cPKC-α redistribution significantly. Restriction of nPKC-ε redistributory activity to staurosporine and selected cogeners suggests that this phenomenon is mechanistically associated with interaction at the catalytic domain of the enzyme, where these agents are thought to inhibit PKC (Tamaoki *et al.*, 1986; Takahashi *et al.*, 1987; Toullec *et al.*, 1991). In contrast, calphostin C (Kobayashi *et al.*, 1989) and miltefosine (Überall *et al.*, 1991) act at the regulatory site. The finding that H-7, which also acts at the catalytic site (Hidaka *et al.*, 1984), did not alter nPKC-ε localisation may be a corollary of its lower affinity for the enzyme compared with that of staurosporine, UCN-01, RO 31-8220 and GF 109203X.

It seems pertinent to compare the concentrations required to elicit nPKC-ε redistribution with those at which these compounds precipitate other biological effects, such as inhibition of PKC activity *in vitro* and arrest of cell growth. The concentrations of UCN-01, RO 31-8220 and GF 109203X which changed nPKC-ε localisation significantly (0.1 μM) exceed their $IC_{50}$ values for PKC inhibition by factors of 3, 4 and 4 respectively. This difference suggests that PKC isoenzyme translocation, which is known to be a consequence of enzyme activation, may also be caused by potent enzyme inhibition. But then it is difficult to explain why CGP 41251, a strong PKC inhibitor, was incapable of causing PKC translocation. Furthermore, in the case of staurosporine the lowest nPKC-ε-redistributory concentrations (1 and 10 nM) were only weakly inhibitory in the PKC activity assay. The concentrations of staurosporine and UCN-01 required for translocation are 2- to 3-fold higher than the $IC_{50}$ values at which they inhibited the growth of A549 cells. Yet in the case of RO 31-8220 and GF 109203X concentrations with antiproliferative efficacy are bound to elicit appreciable translocation of nPKC-ε.

One of the aims of this study was to explore any relationship between structure and activity among staurosporine and four of its analogues with respect to their effects on cell growth and on PKC. In both cell types the order of growth-inhibitory potency was staurosporine > UCN-01 > CGP 41251 > RO 31 8220 > GF 109203X. The order of their enzyme-inhibitory efficacy was RO 31-8220 = GF 109203X = UCN-01 > staurosporine = CGP 41251 with PKC obtained from A549 cells, and staurosporine = UCN-01 = GF 109203X > RO 31 8220 = CGP 41251 with PKC from MCF-7 cells. Intriguingly, CGP 41251 is a strong inhibitor of both cell growth and PKC activity, but incapable of eliciting nPKC-ε translocation. In contrast, UCN-01 interfered strongly with growth and PKC activity and also altered nPKC-ε localisation, whereas RO 31-8220 and GF 109203X are efficacious enzyme inhibitors and caused nPKC-ε redistribution, yet they are rather poor inhibitors of cell growth. These differences in potency allow tentative interpretation of the complicated structure–activity relationships which govern the abilities of these compounds to affect PKC and cell growth. The indolocarbazole staurosporine inhibited PKC and growth with high potency. These properties are not adversely affected by the addition of a hydroxy group at carbon 7 of the aglycone part (UCN-01), or of a benzoyl moiety on the nitrogen in position 4′ of the glycone part of the staurosporine molecule (CGP 41251) (see Figure 1). Yet the presence of *N*-benzoyl abolishes its nPKC-ε translocatory efficacy. The change from the indolocarbazole to the bisindolylmaleimide structural skeleton, that is from staurosporine to RO 31-8220 and GF 109203X, retains the ability to inhibit PKC and to translocate nPKC-ε, but diminishes strongly the growth-inhibitory potential of the molecule.

In conclusion, this study suggests that PKC does not play a direct role in the mechanisms by which PKC inhibitors arrest the growth of A549 and MCF-7 carcinoma cells. Furthermore, it seems that for a PKC inhibitor to affect subcellular enzyme localisation it has to be an analogue of staurosporine which possesses a high affinity for PKC. Further studies on these compounds will help to understand the mechanism by which they interfere with cell growth and perhaps point to other constituents of signal-transducing kinase cascades as suitable targets in the development of novel drugs.

**Abbreviations:** $IC_{50}$, 50% inhibitory concentration; cPKC, conventional ($Ca^{2+}$-dependent) protein kinase C; nPKC, novel ($Ca^{2+}$-independent) protein kinase C; [$^3$H]Tdr, [methyl-$^3$H]thymidine; TPA., 12-*O*-tetradecanoyl phorbor-13-acetate.

**Acknowledgements** This work was supported by a generous grant from the Cancer Research Campaign to Aston University, Birmingham, and to the MRC Toxicology Unit, University of Leicester, and conducted in part in the Pharmaceutical Sciences Institute, Department of Pharmaceutical Sciences, Aston University (Birmingham, UK). We thank Kyowa Hakko Kogyo Co. (Tokyo, Japan) for financial support, Dr P Parker, Imperial Cancer Research Fund Laboratories (London, UK), for the antibody against PKC-ε, and Dr G Pettit, Cancer Research Institute, Arizona State University (Tempe, AR, USA), Dr G Lawton, Roche Research Centre (Welwyn Garden City, UK), Dr T Meier, Ciba Geigy Basle, Switzerland), Dr JA Sullivan, Nova Pharmaceutical Corporation (Baltimore, MD, USA), Dr Y Igarashi, Biomembranes Institute (Seattle, WA, USA), Dr T Tamaoki, Kyowa Hakko Kogyo. (Tokyo, Japan) and Dr P Hilgard, Asta Pharma (Frankfurt, Germany) for samples of bryostatin 1, RO 31-8220, CGP 41251, NPC 15437, trimethylsphingosine, UCN-01 and miltefosine respectively.

### References

AKINAGA S, GOMI K, MORIMOTO M, TAMAOKI T AND OKABE M. (1991). Antitumor activity of UCN-01, a selective inhibitor of protein kinase C, in murine and human tumour models. *Cancer Res.*, **51**, 4888-4892.

BERRA E, DIAZ-MECO MT, DOMINGUEZ I, MUNICIO MM, SANZ L, LOZAN J, CHAPKIN RS AND MOSCAT J. (1993). Protein kinase C ζ isoform is critical for mitogenic signal transduction. *Cell*, **74**, 555-563.

BRADFORD MM. (1976). A rapid and sensitive method for the quantitation of microgram quantities of protein using the principle of protein-dye binding. *Anal. Biochem.*, **72**, 248-254.

BRADSHAW TD, GESCHER A AND PETTIT GR. (1992). Modulation by staurosporine of phorbol-ester-induced effects on growth and protein kinase C localization in A549 human lung carcinoma cells. *Int. J. Cancer*, **51**, 144-148.

CACACE AM, NICHOLS GUADAGNO S, KRAUSS RS, FABBRO D AND WEINSTEIN IB. (1993). The epsilon isoform of protein kinase C is an oncogene when overexpressed in rat fibroblasts. *Oncogene*, **8**, 2095-2104.

DALE IL AND GESCHER A. (1989). Effects of activators of protein kinase C, including bryostatins 1 and 2, on the growth of A549 human lung carcinoma cells. *Int. J. Cancer*, **43**, 158-163.

DALE IL, BRADSHAW TD, GESCHER A AND PETTIT GR. (1989). Comparison of effects of bryostatins 1 and 2 and 12-O-tetradecanoylphorbol-13-acetate on protein kinase C activity in A549 human lung carcinoma cells. *Cancer Res.*, **49**, 3242-3245.

DAVIS PD, ELLIOTT L, HARRIS W, HURST SA, KEECH E, KUMAR H, LAWTON G, NIXON JS AND WILKINSON SE. (1992). Inhibitors of protein kinase C.2. Substituted bisindolylmaleimides with improved potency and selectivity. *J. Med. Chem.*, **35**, 994-1001.

DLUGOSZ AA AND YUSPA SH. (1991). Staurosporine induces protein kinase C agonist effects and maturation of normal and neoplastic mouse keratinocytes *in vitro*. *Cancer Res.*, **51**, 4677-4684.

ENDO K, IGARASHI Y, NISAR M, ZHOU Q AND HAKOMORI S. (1991). Cell membrane signaling as target in cancer therapy: Inhibitory effect of N,N-dimethyl and N,N,N-trimethyl sphingosine derivatives on *in vitro* growth of human tumour cells in nude mice. *Cancer Res.*, **51**, 1613-1618.

GADBOIS DM, HAMAGUCHI JR, SWANK RA AND BRADBURY EM. (1992). Staurosporine is a potent inhibitor of $p34^{cdc2}$ and $P34^{cdc2}$-like kinases. *Biochem. Biophys. Res. Commun.*, **184**, 80-85.

GREIF H, BEN-CHAIM J, SHIMON T, BECHOR E, ELDAR H AND LIVNEH E. (1992). The protein kinase C related PKC-L (η) gene product is localized in the cell nucleus. *Mol. Cell. Biochem.*, **12**, 1304-1311.

GRUNICKE H, HOFMANN J, MALY K, ÜBERALL F, POSCH L, OBERHAMMER H AND FIEBIG H. (1989). The phospholipid- and calcium-dependent protein kinase as target in tumour chemotherapy. *Adv. Enzyme Regulat.*, **28**, 201-216.

HIDAKA H, INAGAKI M, KAWAMOTO S AND SASAKI Y. (1984). 1-(5-Isoquinolinesulfonyl)-2-methylpiperazine (H7), a potent inhibitor of protein kinase C (PKC). *Biochemistry*, **23**, 5036-5041.

HOFMANN J, DOPPLER W, JAKOB A, MALY K, POSCH L, ÜBERALL F AND GRUNICKE H. (1988). Enhancement of the antiproliferative effect of cis-diamminedichloroplatinum (II) and nitrogen mustard by inhibitors of protein kinase C. *Int. J. Cancer*, **42**, 382-388.

ISSANDOU M, BAYARD F AND DARBON JM . (1988). Inhibition of MCF-7 cell growth by 12-O-tetradecanoylphorbol-13-acetate and 1,2-dioctanoyl-sn-glycerol: distinct effects on protein kinase C activity. *Cancer Res.*, **48**, 6943-6950.

KILEY SC, PARKER PJ, FABBRO D AND JAKEN S. (1992). Selective redistribution of protein kinase C isoenzyme by thapsigargin and staurosporine. *Carcinogenesis*, **13**, 1997-2001.

KOBAYASHI E, NAKANO H, MORIMOTO M AND TAMAOKI T. (1989). Calphostin C (UCN-1028C), a novel microbial compound, is a highly potent and specific inhibitor of protein kinase C. *Biochem. Biophys. Res. Commun.*, **159**, 548-553.

MARTINY-BARON G, KAZANIETZ MG, MISCHAK H, BLUMBERG PM, KOCHS G, HUG H, MARME D AND SCHÄCHTELE C. (1993). Selective inhibition of protein kinase C isoenzymes by the indolocarbazole Gö 6976. *J. Biol. Chem.*, **268**, 9194-9197.

MEYER T, REGENASS U, FABBRO D, ALTERI E, RÖSEL J, MÜLLER M, CARAVATTI G AND MATTER A. (1989). A derivative of staurosporine (CGP 41251) shows selectivity for protein kinase C inhibition and *in vitro* anti-proliferative as well as *in vitro* antitumour activity. *Int. J. Cancer*, **43**, 851-856.

MISCHAK H, GOODNIGHT JA, KOLCH W, MARTINY-BARON G, SCHÄCHTELE C, KAZANIETZ MG, MISCHAK H, BLUMBERG PM, PIERCE JH AND MUSHINSKI JF. (1993). Overexpression of protein kinase-δ and -ε in NIH 3T3 cells induces opposite effects on growth, morphology, anchorage dependence, and tumorigenicity. *J. Biol. Chem.*, **268**, 6090-6096.

NISHIZUKA Y. (1992). Intracellular signaling by hydrolysis of phospholipids and activation of protein kinase C. *Science*, **258**, 607-614.

PHILIP PA, REA D, THAVASU P, CARMICHAEL J, STUART NSA, ROCKETT H, TALBOT DC, GANESAN T, PETTIT GR, BALKWILL F AND HARRIS AL (1993). Phase I study of bryostatin 1: assessment of interleukin 6 and tumour necrosis factor α induction *in vivo*. *J. Natl Cancer Inst.*, **85**, 1812-1818.

POSADA JA, MCKEEGAN EM, WOTHINGTON KF, MORIN MJ, JAKEN S AND TRITTON TR. (1989). Human multidrug resistant KB cells overexpress protein kinase C; involvement in drug resistance. *Cancer Commun.*, **1**, 285-292.

PRENDEVILLE J, CROWTHER D, THATCHER N, WOLL PJ, FOX BW, MCGOWN A, TESTA N, STERN P, MCDERMOTT R, POTTER M AND PETTIT GR. (1993). A phase I study of intravenous bryostatin 1 in patients with advanced cancer. *Br. J. Cancer*, **68**, 418-424.

SATO W, YUSA K, NAITO M AND TSURUO T. (1990). Staurosporine, a potent inhibitor of C-kinase, enhances drug accumulation in multidrug-resistant cells. *Biochem. Biophys. Res. Commun.*, **173**, 1252-1257.

SCHWARTZ GK, JIANG J, KELSEN D AND ALBINO AP. (1993). Protein kinase C: a novel target for inhibiting gastric cancer cell invasion. *J. Natl Cancer Inst.*, **85**, 402-407.

STANWELL C, GESCHER A, BRADSHAW TD AND PETTIT GR. (1994). The role of protein kinase C isoenzymes in growth inhibition caused by bryostatin 1 in human A549 lung and MCF-7 breast carcinoma cells. *Int. J. Cancer*, **56**, 585-592.

SULLIVAN JP, CONNOR JR, SHEARER BG AND BURCH RM. (1991). 2, 6-Diamino-N-([1-(1-oxotridecyl)-2-piperidinyl]methyl)hexanamide (NPC15437): a selective inhibitor of PKC. *Agents Actions*, **34**, 142-144.

TAKAHASHI I, KOBYASHI E, ASANO K, YOSHIDA M AND NAKANO H. (1987). UCN-01, a selective inhibitor of protein kinase C from streptomyces. *J. Antibiot.*, **40**, 1782-1784.

TAMAOKI T AND NAKANO H. (1990). Potent and specific inhibitors of protein kinase C of microbial origin. *Biotechnology*, **8**, 732-735.

TAMAOKI T, NOMOTO H, TAKAHASU I, KATO Y, MORIMOTO M AND TOMITA F. (1986). Staurosporine, a potent inhibitor of protein kinase C from streptomyces. *Biochem. Biophys. Res. Commun.*, **135**, 397-402.

TOULLEC D, PIANETTI P, COSTE H, BELLEVERGUE P, GRAND-PERRET T, AJAKANE M, BAUDET V, BOISSIN P, BOURSIER E, LORIOLLE F, DUHAMEL L, CHARON D AND KIRILOVSKY J. (1991). The bisindolylmaleimide GF 109203X is a potent and selective inhibitor of protein kinase C. *J. Biol. Chem.*, **266,** 15771-15781.

ÜBERALL F, OBERHUBER H, MALY K, ZAKNUN J, DEMUTH L AND GRUNICKE HH. (1991). Hexadecylphosphocholine inhibits inositol phosphate formation and protein kinase C activity. *Cancer Res.*, **51,** 807-812.

UTZ I, HOFER S, REGENASS U, HILBE W, THALER W, GRUNICKE H AND HOFMANN J. (1994). The protein kinase C inhibitor CGP 41251, a staurosporine derivative with antitumor activity, reverses multidrug resistance. *Int. J. Cancer*, **57,** 104-110.